# Prevalence and factors associated with intestinal parasites among food handlers in Medebay Zana District, north West Tigray, northern Ethiopia

**DOI:** 10.1186/s40794-020-00123-1

**Published:** 2021-01-31

**Authors:** Kebrom Regassa, Kiros Tedla, Gessessew Bugssa, Gebretsadkan Gebrekirstos, Hailay Gebreyesus, Mebrahtu Teweldemedhin Shfare

**Affiliations:** 1 Department of Medical Parasitology and Entomology, College of Health Science, Mekelle University, Mekelle, Ethiopia; 2 College of Health Sciences, Aksum University, Aksum, Tigray Ethiopia

**Keywords:** Intestinal parasites, Food handlers, Ethiopia

## Abstract

**Introduction:**

Intestinal parasites are amongst the major public health challenges facing Sub-Saharan Africa. The aim of the study was to determine the prevalence of intestinal parasites and its associated factors among food handlers in Medebay Zana District, Tigray, Ethiopia.

**Methods:**

A cross-sectional study was conducted among 401 food handler individuals selected by systematic random sampling. Binary and multivariable logistic regression was used to determine the possible association between the independent variable and outcome variables. Statistical significance was declared at *p*-value < 0.05 with a 95% confidence interval.

**Result:**

The prevalence of intestinal parasites was 33.2% within this sample. The dominant parasite was *Entamoeba coli* 50(37.4%), followed by *Entamoeba histolytica/dispar* 24(18%), *Entamoeba hartmanni* 18(13.5), *Giardia lamblia* 17(12.8%), *Schistosoma mansoni* 8(6%), *Hymenolepis nana* 7(5.3%), *Entervious vermicularies* 6(4.5%) and *Taenia species* 3(2.5%).

**Conclusion:**

This study revealed a high prevalence of intestinal parasites among food handlers for a range of intestinal parasites. The significant predictors were the source of water, washing hands before food preparation, washing hands with soap and water after visiting the toilet, shower installation at the workplace, washing the body regularly and eating raw vegetables and raw meat. Hence, local health planners should implement appropriate interventional measures for the novel risk factors to mitigate the problem.

**Supplementary Information:**

The online version contains supplementary material available at 10.1186/s40794-020-00123-1.

## Introduction

Infection with intestinal parasites remains a major public health challenge in developing countries where poor environmental sanitation, poor personal hygiene, and a low level of education are prominent [1]. Globally, about one-third of the total population is estimated to be infected with intestinal parasites, the majority being people living in tropical and sub-tropical parts of the world [2]. The World Health Organization (WHO) estimated that at least one-quarter of the world’s population is infected with soil-transmitted helminths [3]. Worldwide, about 300 million people suffer from severe helminthic infections, leading to morbidity and over 150,000 deaths annually [4]. The reasons for the incidence of parasites include climatic conditions, local traditions, and the use of human and animal fertilizers in agriculture and vegetable planting [5]. Food sold in markets may be contaminated by hands that have not been washed after defecation or from carriers that land on both food and feces, thereby increasing the risk of transmission of intestinal parasites to consumers [6].

Regarding the etiologies, *Ascaris lumbricoides*, *Trichuris trichiura*, *Hook worm*, *Entamoeba histolytica*, and *Giardia lamblia* were the common causes [7]. As in many developing countries, cases of intestinal parasites are highly abundant in Ethiopia. The country has the second-highest burden of Ascariasis, the third highest burden of hookworm, and the fourth highest burden of Trichuriasis in Sub-Saharan Africa [8].

Industrialization and urbanization have prompted people to migrate from rural to urban areas, forcing them to have their meals at any place at an affordable price. In urban areas, there is mushrooming of eating establishments due to increased demand with the concomitant potential to spread of disease via food handlers [9].

The epidemiology of intestinal parasites remained complex because of the diversity of associated factors involved, with the complexities of control highly relevant to addressing this escalating challenge [10]. In Ethiopia, particularly in the study area, intestinal parasitosis is a steadily increasing public health concern [11, 12]. On the other hand, the study area is not researched and there is a paucity of scientific information in the region at large. Therefore, this cross-sectional study was conducted to assess the prevalence and associated factors of intestinal parasites among food handlers in Medebay Zana district Towns, Tigray, Ethiopia which can be an input for timely interventional measures on the actual risk factors.

## Methods and materials

### Study area, design, and period

The cross-sectional, community-based study was conducted among food handlers working in food service establishments in Medebay Zana district Towns from January 2019 to February 2019. Medebay Zana district is about 1040 km from Addis Ababa. In the district, there were a total of 277 restaurants and hotels, 44 snack bars, and 52 food bakeries. These establishments can accommodate a total of 1850 people. In total, in the hotels, restaurants, and snack bars found in the district, there are 1050 food handlers.

### Population

All food handlers who were working in Medebay Zana district Towns were the target population; randomly selected (see the sampling technique) food handlers formed the study population. Food handlers who were under treatment or those who finished treatment for parasitic infection within 2 weeks prior to the study were excluded.

### Sample size and sampling technique

The sample size was determined using the following assumptions (level of confidence of the population is taken to be 95% and Zα/2 = 1.96). A 5% margin of error (d = 0.05) and the prevalence of intestinal parasites among food handlers were based on a previously conducted study in Ethiopia, Tigray Mekelle Town at 49.3% [11]. Based on these assumptions, the actual sample size for the study is computed using one sample population proportion formula as indicated below.

$$ \mathbf{n}={\left(\mathbf{Z}\boldsymbol{\upalpha } /\mathbf{2}\right)}^{\mathbf{2}}\mathbf{P}\ \left(\mathbf{1}-\mathbf{P}\right)/{\mathbf{d}}^{\mathbf{2}} $$

Then, the sample size was, **n ≈ 384** + 10% contingency = **422**. The total sample size was proportionally allocated according to the number of food handlers working in hotels, snack bars, and bakeries; a systematic random sampling technique was then employed to select the allocated number of study participants from the sampling frame.

### Data collection tools

Interviewers administered structured questionnaires were used to collect the data. The content of the questionnaire included socio-demographic characteristics, educational status, economic status, individual behavioral factors, and house characteristics as well as environmental conditions.

### Laboratory investigation

Fecal specimens were obtained from the selected food handlers and transported to the laboratory-based on the standard operating procedures for the collection and transportation of fecal specimens. In the laboratory, the stool was immediately examined by wet mount technique for motile parasites, helminth eggs, cysts, and oocysts of intestinal protozoa followed by Kato-Katz and formal ether concentration technique [13].

### Study variables

#### Dependent variable

Intestinal parasites status.

#### Independent variable

**Socio-demographic variables,** such as age, sex, monthly income, educational level, service year; **Individual factors,** such as proper personal hygiene practice, using fresh vegetable salads and meat, use of sanitizer and disinfectants, environmental sanitation, contact with a water source, contact with an animal, eating raw meat and vegetables; **Economic factors,** such as quality of housing, isolation from work environment when sick, lack of supply of safe water, lack of access to toilet facility, lack of medical check screening.

### Data quality assurance

To assure the data quality, the data collection instrument was pretested in nearby towns. The data collectors were trained before the actual data collection period by experts from Tigray Health and Research Institute. Furthermore, the collected data were reviewed and checked for completeness each day. Laboratory investigations were done as per the standard operating procedures for specimen collection, transportation, and analysis of fecal specimens.

### Data analysis

After checking for completeness, data were coded, cleaned, and entered into SPSS version 21.0 for analysis. Binary logistic regression was used to determine the possible associations between the independent variables and an outcome variable. Those factors with a *p*-value of 0.2 and below during the bivariate logistic regression analysis were considered for multivariate logistic regression to control the confounders. Finally, statistically significant was declared at p-value < 0.05 with a 95% confidence interval.

## Result

### Socio-demographic characteristics

From the total of 422 study participants, 401 were included in this research (giving a 95% response rate. Out of the 401 participants, 377(94.0%) were females, and the mean age of all study participants were 28.74(±SD = 4.56). From the total participants, 21(5.2%) have completed college and above, 78(19.5%) completed secondary school, 273(68.1%) completed primary school and the remaining 29(7.2%) were unable to read and write. The study also showed that 212 (52.9%) participants had monthly incomes of between 500 and 900 Ethiopian birr and the remaining 189 (47.1%) participants earned ≥1000 birr monthly (Table 1).

**Table 1:** Socio-demographic characteristics of study participants (*n* = 401) among food handlers in Medebay Zana district (February–March, 2019)

Characters	Category	Frequency	Percent
Sex	Female	377	94.0
	Male	24	6.0
Age	**<** 21	28	7.0
	21–30	254	63.3
	31–40	110	27.4
	> 40	9	2.2
Religious	Orthodox	398	99.3
	Muslim	3	0.7
Educational leve	College and above	21	5.2
	Secondary school	78	19.5
	Primary school	273	68.1
	Unable to read and write	29	7.2
Monthly income	500–900 birr	212	52.9
	1000–1500 birr	189	47.1
Service in year	1–5 year	332	82.8
	6–9 year	69	17.2

### Hygiene related factors of food handlers

According to the responses, 306(76.3%) study participants have washed their hands before food preparation, 73(18.2%) washed their hands usually, and 22(5.5%) washed their hands sometimes. Out of the total participants, 222(55.4%) washed their hands with soap and water after using the toilet, 378(94.3%) washed their hands after touching dirty materials and different body parts, 180(44.9%) washed their body regularly in their working area, and 284(70.8%) participants had renewed their medical certificate every 3 months (Table S1).

### Working area related factors of food handlers

From the total of 401 study participants, 386 (96.3%) used private tap water, 349(87%) had a shower facility in their workplace, 287(71.6%) had separate dressing rooms in their working area, 310(77.3%) used water and detergent to clean utensils and drinking cups and 87(21.7%) cleaned the kitchen floor at least three times per day (Table S2).

### Prevalence and type of intestinal parasite

Based on a microscopic stool sample examinations, eight species of intestinal parasites were identified with an overall prevalence of 33.2%. From the total species of intestinal parasites detected the most prevalent was protozoan 109(81.7%) followed by helminths 24(18.3%) (Table 2).

**Table 2:** Prevalence and type of intestinal parasites among the positive participants in District Medebay Zana district towns (February–March, 2019)

Parasites category	Number (%)
**Protozoan**	109(81.7)
*Entamoeba coli*	50(37.4)
*Entamoebahistolytica/dispar*	24(18)
*Entamoeba hartmanni*	18(13.5)
*Giardia lamblia*	17(12.8)
**Helminths**	24(18.3)
*Schistosoma mansoni*	8(6.0)
*Hymenolepis nana*	7(5.3)
*Entervious vermicularies*	6(4.5)
*Taenia species*	3(2.3)
**Total**	133(100%)

### Factors associated with the occurrence of intestinal parasites

The findings from the bivariate analysis showed that 13 variables meet the criteria of p-value < 0.2 to be included for multivariate analysis (Table 3). From the total of 13 variables that met the criteria only 7 variables were significantly and independently associated with the occurrence of intestinal parasites at a *p* < 0.05 and the 95% confidence interval. The analysis from the multivariate logistic regression showed that food handlers who have the habit of eating raw vegetables and meats were 31.9 times more likely to be positive for intestinal parasites compared to their counterparts [AOR = 31.92, 95% CI = 10.01–101.80]. Additionally, food handlers that use public tap water were 11.5 times more likely to be positive for intestinal parasites compared to those that used private tap [AOR = 11.59, 95% CI = 1.73–77.45]. Those food handlers who washed their hands sometimes before food preparation was 3.25 times more likely to be positive for intestinal parasites compared to those who washed always [AOR = 3.25, 95% CI = 1.33–7.92]. Those food handlers who washed their hands sometimes with soap and water after visiting the toilet were 3.54 times more likely to be positive for intestinal parasites compared to those who washed their hands always [AOR = 3.54,95% CI = 1.72–7.31]. Similarly, food handlers who have no shower facility at the workplace were 3.84 times more likely to be positive for intestinal parasites compared to those who have a shower facility [AOR = 3.84, 95% CI = 1.47–10.26].

**Table 3:** Factors associated with the occurrence of intestinal parasite from the results of bivariate and multivariate logistic regression analysis among the study participant in Medebay Zana district Towns (February–March, 2019)

Variables	Category	Intestinal parasite		COR (CI)	AOR (CI)
		No (%)	Yes (%)		
What is the source of water in your working area?	Public tap	4(26.7%)	11(73.3%)	5.95(1.86–19.06)	11.59(1.73–77.45)^a^
	Private tap	264(68.4%)	122(31.6%)	1	1
How often you wash your hands before food preparation?	Sometimes	8(36.4%)	14(63.6%)	5.95(1.91–5.48)	3.25(1.33–7.92)^a^
	Usually	34(46.6%)	39(53.4%)	4.95(1.99–12.22)	4.12(1.13–15.05)^a^
	Always	226(73.9%)	80(26.1%)	1	1
Do you wash your hands by soap and water after visiting toilet?	Sometimes	6(18.8%)	26(81.2%)	5.45(3.34–8.88)	3.54(1.72–7.31)^a^
	Usually	74(50.3%)	73(49.7%)	23.96(9.17–62.57)	6.05(1.67–21.92)^a^
	Always	188(84.7%)	74(15.3%)	1	1
Have you used your sick leave properly to obtain treatment?	No	56(47.9%)	61(52.1%)	3.21(2.04–5.03)	
	Yes	212(74.6%)	72(25.4%)	1	
Does your workplace have shower facility?	No	18(34.6%)	34(65.4%)	4.77(2.57–8.84)	3.84(1.47–10.26)^a^
	Yes	250(71.6%)	99(28.4%)	1	1
Do you wash your body regularly in your working area?	No	116(52.4%)	105(47.5%)	4.91(3.03–7.95)	2.48(1.09–5.61)^a^
	Yes	152(84.4%)	28(15.6%)	1	1
Do you have habit to eat raw vegetables &meat?	Yes	6(12.8%	41(87.2%)	19.46(7.99–47.34)	31.92(10.01–101.80)^a^
	No	264(74.0%	92(26%)	1	1
Does your working area have separate dressing room?	No	55(48.2%)	59(51.8%)	3.08(1.96–4.85)	
	Yes	213(74.2%)	74(25.8%)	1	
Do you wear clean aprons when you preparing food?	No	63(46.0%)	74(54%)	4.08(2.61–6.36)	
	Yes	205(77.7%)	59(22.3%)	1	
Do you wear hair garments during preparing food?	No	59,948.4%)	63(51.6%)	3.18(2.04–4.98)	
	Yes	209(74.9%)	70(25.1%)	1	
How frequently the kitchen floor cleaned? (per day)	One times	35(30.4%)	80(69.6%)	30.85(12.30–77.38)	13.73(4.21–44.77)^a^
	Two times	152(76.4%)	47(23.6%)	4.17(1.71–10.18)	2.51(0.85–7.39)
	Three times	81(93.1%	6(6.9%)	1	1
Monthly income (birr)	500–9,001,000 –	119(56.1%)	93(43.9%)	2.91(1.87–4.52)	
	1500	149(78.8%)	40(21.2%)	1	

Note: *CI* Confidence interval, *AOR* Adjusted odds ratio, *COR* Crude odds ratio ^a^ = Significant associated factors

## Discussion

The study researched unaddressed areas in the Tigray region and generated new findings with regard to the prevalence of intestinal parasites among food handlers and the potential risk factors. The findings will have paramount importance for the evidence-based interventions that ensure food safety, improve the health of food handlers and the public at large. In this cross-sectional study, the overall prevalence of intestinal parasites among food handlers was 33.2% in Medebay Zana District, North West Tigray. The prevalence of intestinal parasites in this study were relatively higher than the studies reported in Ethiopia, Aksum Town 14.5% [14], Gondar, Northwest Ethiopia 29.1% [15], North East India 29.33% [16], Saudi Arabia 23% [17], Accra, Ghana at 21.6% [18], Kenya 23.7% [19], Omdurman, Sudan 6.9% [20], Turkey 8.8% [21], and Sari and Northern Iran 15.5% [22]. Whereas lower than the studies done in South West Ethiopia 44.1% [7], Bahr Dar Town 41.1% [23], Jima Town 48.2% [24], Tigray Mekelle Town 49.3% [11], and India Tertiary Care Hospital 40.5% [25].The prevalence of intestinal parasites in the current study was consistent with the studies conducted in Southern, Ethiopia which were 36% [26] and a study conducted in Qatar reporting 33.9% prevalence [27].

The variability from our current prevalence may reflect differences in socioeconomic status, climatic conditions, poverty, personal and community hygiene, and period of the study. Differences in the study time might have also contributed because our study was conducted during the time when irrigational movement was high; hence, the entire data set was collected during dry months which may contribute to raising the prevalence of schistosomiasis and other helminths. In many arid or semi-arid habitats, some protozoan parasites are common around the end of the rainy season [20]. The cultural habit of eating raw meat in some areas might have also contributed to the increasing presence of helminths [5].

In the present study, many intestinal parasitic infections were found with *S. mansoni* being the predominant parasite from helminths followed by Hymenolepis nana. This finding was relatively consistent with a similar study conducted in Aksum Town, Ethiopia [14]. *Entamoeba coli* was the most predominant parasite from protozoan parasites followed by *E.histolytica/dispar* which resembled findings from a similar study conducted in Southern Ethiopia, reporting the prevalence of (36%) [26].

Poor personal and environmental hygiene, including using public tap water and eating raw vegetables and meat among food handlers is a common practice that contributes to food and water-borne as well as skin transmitted diseases [11]. Parasite eggs in the environment can contaminate water source, vegetables, hands and subsequently ingested with foods [22]. Hence, in this study, the untrimmed source of water, washing hands before food preparation, washing hands with soap and water after visiting the toilet, shower facilities in the workplace, regular washing of one’s body, habit to eat raw vegetables & meat and kitchen floor cleaned were identified as determinant factors for intestinal parasites among food handlers. In comparison with other studies, poor hand washing practice was similarly found to be a significant predictor of intestinal parasitosis among food handlers in Southwest Ethiopia [7].

## Conclusion

In conclusion, this study revealed a high prevalence of intestinal parasite among food handlers who tested positive for different intestinal parasites. Source of water, hand washing after visiting the toilet and before food preparation, shower facilities, washing one’s body regularly, practices related to eating raw vegetables and meat, and kitchen floor cleaned were the identified factors affecting food handlers for the risk of acquiring the intestinal parasite in the study area. Hence, the existing health extension package interventional measures particularly on improving personal hygiene and environmental sanitation should be strengthened and sustained in order to further reduce intestinal parasitic infections among food handlers and the community at large. Comprehensive community-based health education should be given for food handlers targeting on risk factors of intestinal parasite. This study was conducted within a single district on limited numbers of participants who were working in foodservice establishments which mean the findings may not be generalizable to the overall food handlers working in the region. Thus we also recommend researchers to conduct large scale follow up studies in a regional context.

## Supplementary Information

Additional file 1: **Table S1.** Personal hygiene characteristics of study participants (*n* = 401) among food handlers in MedebayZanadistrict Towns (February–March, 2019).

Additional file 2: **Table S2.** Working area related factors of food handlers (*n* = 401) in MedebayZana district Towns (February–March, 2019).

## Data Availability

All data generated or analyzed during this study is included in this published article.
