# Supplementary material for: Prevalence and factors associated with intestinal parasites among food handlers in Medebay Zana District, north West Tigray, northern Ethiopia
Source: Trop Dis Travel Med Vaccines. 2021 Jan 31;7:2. doi: 10.1186/s40794-020-00123-1 (PMC7847587; doi:10.1186/s40794-020-00123-1)
Supplement: Supplementary file 1 — Table S1. Personal hygiene characteristics of study participants (n = 401) among food handlers in MedebayZanadistrict Towns (February–March, 2019). [file 40794_2020_123_MOESM1_ESM.docx]

**Table S1: Personal hygiene characteristics of study participants (n=401) among food handlers in MedebayZanadistrict Towns (February-March, 2019).**

| Characters | Category | Frequency | Percent |
| --- | --- | --- | --- |
| How often do you wash your hands before food preparation? | Always  Usually  Sometimes | 306  73  22 | 76.3  18.2  5.5 |
| Do you wash your hands by soap and water after visiting toilet? | Always  Usually  Sometimes | 222  147  32 | 55.4  36.7  8.0 |
| Do you wash your hands after touching dirty materials and different body parts? | No  Yes | 23  378 | 5.7  94.3 |
| Do you wash your body regularly in your working area? | No  Yes | 221  180 | 55.1  44.9 |
| Do you have a medical checkup certificate? | No  Yes | 0  401 | 0.0  100 |
| How frequent do you come to health center for medical checkup? | Every three month  Every six month  Every nine month | 284  70  47 | 70.8  17.5  11.7 |
| Do you wear clean aprons during food preparation? | N0  Yes | 137  264 | 34.2  65.8 |
| Do you wear clean hair garment during food preparation? | No  Yes | 122  279 | 30.4  69.5 |
| How frequent do you cut your fingernails? | Two times per week  One times per week  One times per two week | 29  75  295 | 7.2  18.2  74.1 |
