# Supplementary material for: Prevalence and factors associated with intestinal parasites among food handlers in Medebay Zana District, north West Tigray, northern Ethiopia
Source: Trop Dis Travel Med Vaccines. 2021 Jan 31;7:2. doi: 10.1186/s40794-020-00123-1 (PMC7847587; doi:10.1186/s40794-020-00123-1)
Supplement: Supplementary file 2 — Table S2. Working area related factors of food handlers (n = 401) in MedebayZana district Towns (February–March, 2019). [file 40794_2020_123_MOESM2_ESM.docx]

Table S2: Working area related factors of food handlers (n =401) in Medebay Zana district Towns (February-March, 2019)

| Variables | Category | Frequency | Percent |
| --- | --- | --- | --- |
| What is the source of water in your working area? | Private tap  Public tap | 386  15 | 96.3  3.7 |
| Does your working area have shower facility? | No  Yes | 52  349 | 13  87 |
| Does your working area have separate dressing room? | No  Yes | 114  287 | 28.4  71.6 |
| What do you use to clean utensils and drinking cup? | Only water  Water and detergent  Hot water and detergent | 53  310  38 | 13.2  77.3  9.5 |
| How frequently is the kitchen floor cleaned? | One times per day  Two times per day  Three times per day | 115  199  87 | 28.7  49.6  21.7 |
| Do you have toilet facility in your working area? | No  Yes | 0  401 | 0  100 |
